# Electrophysiological assessment of radial shock wave therapy for carpal tunnel syndrome

**DOI:** 10.3389/fnins.2023.1251807

**Published:** 2023-10-31

**Authors:** Ya Zong, Hong Zhang, Peipei Xu, Maoqi Chen, Qing Xie, Ping Zhou

**Affiliations:** ^1^Department of Rehabilitation, Ruijin Hospital, Shanghai Jiao Tong University School of Medicine, Shanghai, China; ^2^School of Rehabilitation Science and Engineering, University of Health and Rehabilitation Sciences, Qingdao, Shandong, China

**Keywords:** carpal tunnel syndrome (CTS), shock wave therapy, compound muscle action potential (CMAP) scan, motor unit number estimation (MUNE), MScanFit, StairFit, step index (STEPIX)

## Abstract

This study presents an electrophysiological assessment of radial extracorporeal shock wave therapy on patients with carpal tunnel syndrome (CTS). Sixteen CTS subjects received radial extracorporeal shock wave therapy once a week for five consecutive weeks. Outcome performance was assessed using the Boston Carpal Tunnel Questionnaire (BCTQ) and electrodiagnostic measurements including a nerve conduction study of the median nerve and a compound muscle action potential (CMAP) scan of the abductor pollicis brevis muscle. The BCTQ and the sensory conduction test measurements were all statistically improved after the treatment. However, the motor conduction test measurements were not significantly different before and after the treatment. The CMAP scan examination revealed MScanFit motor unit number estimation (MUNE) was significantly higher after the treatment, while no significant change was found in StairFit MUNE and step index. These results confirmed the effectiveness of shock wave therapy for treating CTS symptoms and the associated sensory property changes. The reasons for the inconsistencies from different CMAP scan processing methods are worthwhile targets for further investigation.

## 1. Introduction

Carpal tunnel syndrome (CTS) is one of the most common disorders of peripheral nerve entrapment syndrome (Wipperman and Goerl, 2016). Patients with CTS often suffer from hand numbness, pain, muscle atrophy and weakness due to compression of the median nerve at the wrist as it passes through the carpal tunnel, which is a fibrous canal composed of the carpal bones and flexor support bands (nerves and tendons). Early symptoms manifest as numbness and tingling of the fingers on the radial side of the hand. Aggravated symptoms can cause pain and result in frequent waking at night, and may also develop progressive muscle atrophy and the inability to hold objects. One study reported that 90% of median neuropathies are caused by CTS, with a morbidity rate up to 3.8% and a higher prevalence in women than in men (Aboonq, 2015). CTS often occurs in people who require frequent use of wrist movements such as computer operators, dishwashers, and carpenters. CTS can also be complicated by pregnancy, diabetes or trauma to the carpal tunnel.

The treatment of CTS can use both conservative and surgical approaches. The former incudes wrist splinting, local corticosteroid injections, laser therapy, and therapeutic ultrasound, etc. Most conservative treatments are slow to work and usually for those patients with relatively mild symptoms. In contrast, surgical treatments can target more severe patients but are traumatic and leave scars. Of note, about 60–70% of conservative treatments remain symptomatic for about 18 months (Katz et al., 1998). Surgical treatments also have a failure rate of approximately 7% and a recurrence of symptoms in approximately 75% of patients (Akhtar et al., 2007). These highlight the importance of developing new treatment modalities. For example, a recent study has reported ultrasound-guided needle release plus corticosteroid injection for improving treatment of CTS (Zeng et al., 2023).

Radial extracorporeal shock wave therapy has been used clinically to treat aseptic soft tissue inflammatory diseases such as plantar fasciitis. Shock waves are a series of acoustic pulses characterized by high and rapid peak pressure, which can propagate in three-dimensional space. In recent years, radial shock wave therapy has been used as a convenient and non-invasive treatment for CTS and has demonstrated positive clinical results (Wu et al., 2016; Xie et al., 2022). The extracorporeal shock waves used for treatment are usually low energy and result in minimal side effects or risk of complications (Rompe et al., 1998). Typical outcome assessment of CTS treatments mainly relies on clinical scales, which are often subjective. In contrast, objective evaluation of neuromuscular changes following treatment is lacking in common practice.

In this study, shock wave therapy was used to treat CTS, which was subsequently evaluated by both clinical scales and electrophysiological examinations. In addition to routine nerve conduction studies, compound muscle action potential (CMAP) scans were recorded before and after the treatment (Visser and Blok, 2009). Motor unit number changes were examined using different CMAP processing methods including MScanFit motor unit number estimation (MUNE) (Bostock, 2016; Jacobsen et al., 2018), StairFit MUNE (Chen et al., 2023), and step index (STEPIX) (Nandedkar et al., 2022). To the best of our knowledge, this is the first CMAP scan study in CTS population. We hypothesize that shock wave therapy can help functional improvement of CTS patients, evidenced by BCTQ and electrophysiological parameters.

## 2. Methods

### 2.1. Subjects

Subjects who had a definite diagnosis of CTS were recruited. Diagnosis followed the electrophysiological diagnostic and severity criteria for CTS recommended by the American Association of Neuromuscular & Electrodiagnostic Medicine (Werner and Andary, 2011). Subjects with other diseases such as cervical radiculopathy and multiple peripheral neuropathies were excluded from the study. The study was approved by the Institutional Review Board of Shanghai Jiao Tong University. All subjects gave informed consent before participating in the study.

A total of 16 subjects (2 males, 14 females) were recruited. All subjects were right-handed. Their mean age was 54.8 ± 13.6 years (range: 37 to 84 years), mean height was 159.6 ± 6.5 cm (range: 150 to 177 cm), mean duration of the disease was 45.1 ± 46.1 months (range: 1 to 180 months). Five subjects had unilateral CTS on the right side and 11 subjects had bilateral CTS. Among 11 bilateral subjects, the right side was more severe (demonstrated as longer latency and/or lower sensory or CMAP amplitude) in 7 subjects and the left side was more severe in 4 subjects. The more severe side was examined for all bilateral CTS subjects except for 1 subject who did not elicit a clear waveform for motor conduction and therefore the other side was examined for this subject.

### 2.2. Radial shock wave therapy

**Figure 1:**
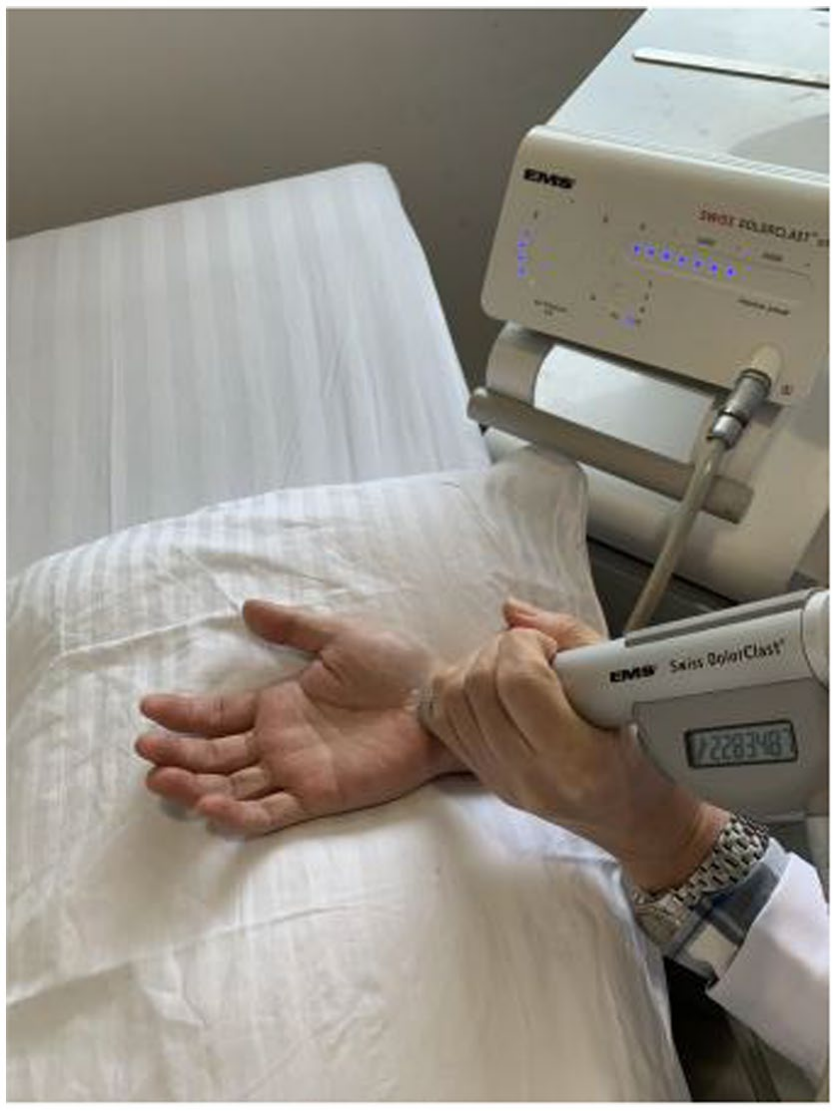
Schematic illustration of the radial shock wave therapy on the carpal tunnel of a CTS subject.

The patients were treated with radial shock wave therapy once a week for five consecutive weeks. A Swiss EMS Extracorporeal Discharge Shockwave Therapy device (Swiss DolorClast Classic, EMS Electro Medical Systems S. A., Nyon, Switzerland) was used. The probe was placed on the carpal tunnel as treatment site (Figure 1). The shock wave treatment included 1,500 shots at a frequency of 10 Hz, and a pressure of 1.5 Bar. These parameters were determined by collectively considering previous literatures (Ke et al., 2016; Atthakomol et al., 2018; Gholipour et al., 2023; Menekseoglu et al., 2023) and subject feedback from a preliminary testing. During the treatment, the operator slowly moved the probe within the treated area. Pain medication and anesthesia were not applied as no subject had pain complaints during the treatment. Both clinical scales and electrophysiological examinations were performed before the first treatment and 1 week after the last treatment, which are detailed in the following section.

### 2.3. Outcome measurements

#### 2.3.1. BCTQ

The Boston Carpal Tunnel Questionnaire (BCTQ) was used to assess the qualitative improvements after the treatment (Levine et al., 1993). The BCTQ consists of a two-part questionnaire: 11 items for the symptom severity scale and 8 items for the functional capacity scale. The symptom severity scale assesses the impact or severity of CTS-related symptoms (e.g., wrist pain, numbness, its frequency and duration, whether it interfered with night sleep, the number of numb awakenings, and whether it was difficult to grasp small objects, etc.). The functional capacity scale assesses the ability or difficulty of common activities of daily living (e.g., bathing, dressing, writing, household chores, gripping of a telephone, etc.). Higher scores indicate the symptoms and impaired functional capacity are more severe.

#### 2.3.2. Nerve conduction studies

Both motor and sensory conduction studies were performed for the median nerve innervating the abductor pollicis brevis (APB) muscle. The testing room temperature was set 28–30°C, and the subject’s skin temperature was maintained above 32°C. The study was performed with a Nicolet EDX EMG system (Natus Neurology Incorporated, Middleton, WI, United States). A standard bar stimulating surface electrode was used which has two contact surfaces 20 mm apart, 9 mm in diameter for each. The active and the reference electrodes were disposable Ag–AgCl surface electrodes, 13 mm in diameter.

##### 2.3.2.1. Motor conduction study

The recording electrode was placed on the APB muscle belly, and the reference electrode was placed at the metacarpophalangeal joint of the ipsilateral thumb. The ground electrode was placed at the dorsum of the ipsilateral hand. For electrical stimulation, the distal end stimulation was applied to the median nerve between the flexor carpi radialis tendon and the palmaris longus tendon at the transverse wrist, and the proximal end stimulation was applied to the median nerve between the long head of the biceps muscle and the medial epicondyle of the humerus at the elbow. CMAP amplitude, motor latency and motor nerve conduction velocity were examined.

##### 2.3.2.2. Sensory conduction study

The recording electrode was placed at the root of the index finger on the subject’s examined side, and the reference electrode was placed at approximately 4 cm from the recording electrode at the distal end of the ipsilateral index finger. The grounding electrode was placed on the back of the ipsilateral hand. Electrical stimulation was applied to the median nerve at a distance of approximately 13 cm from the recording electrode between the radial carpal flexor tendon and the palmaris longus tendon at the transverse wrist. Sensory action potential amplitude, latency and conduction velocity were examined.

#### 2.3.3. CMAP scan examination

CMAP scan recording: The active, reference and ground electrode placement was similar to motor conduction study. The median nerve was stimulated between the flexor carpi radialis tendon and the palmaris longus tendon at the transverse wrist stripe. The stimulation current intensities S0 and S100 were first determined. S0 represents the lowest intensity to activate the first motor unit, and S100 represents the highest intensity required to activate all motor units. After setting the stimulation range, the CMAP scan was performed using a protocol of 500 stimuli at a frequency of 2 Hz, with intensity linearly declined from highest to lowest. The stimulus pulse width was 0.1 ms.

CMAP scan processing: To evaluate motor unit number alteration, the CMAP scan data was processed using three different methods, namely MScanFit MUNE (Bostock, 2016; Jacobsen et al., 2018), StairFit MUNE (Chen et al., 2023) and STEPIX (Nandedkar et al., 2022). The default setting of model options (relative spread RS% = 2, deleted units <25 μV applied, set number of units to 20) was used when implementing MScanFit MUNE. For StairFit MUNE, the threshold was set as 15 μV.

### 2.4. Statistical analysis

Paired t-test was applied to compare the normally distributed parameters before and after the treatment. Wilcoxon signed rank test was applied if the data was non-normally distributed parameters (including S100-S0, BCTQ, assessed by Shapiro–Wilk test). All the analysis was performed using MATLAB (MathWorks Inc., Natick, MA). Significance level was defined as *p* < 0.05.

## 3. Results

All subjects completed the scheduled treatment and testing. No local tissue effects, pain, bleeding, or other complications were reported. Table 1 summarizes a comparison of outcome and electrophysiological measurements before and after the treatment. It can be observed from the table that the BCTQ scale was significantly reduced after the treatment. The differences in latency, potential amplitude, and conduction velocity in the sensory conduction study of the median nerve were statistically significant before and after the treatment. However, the parameters of the motor conduction test were not significantly changed. For the CMAP scan examination, the motor unit number estimated from the MScanFit program was significantly higher after the treatment. An associated finding was that the mean motor unit size derived from MScanFit was significantly smaller after the treatment. In contrast, there was no significant difference in the motor unit number or mean motor unit size estimated from Stairfit MUNE and STEPIX before and after the treatment. There was no significant difference in S0, S100, and the stimulation range (S100–S0) before and after the treatment.

**Table 1:** A comparison of different parameters of CTS subjects before and after shock wave therapy treatment.

		Pre-treatment	Post-treatment	
BCTQ		29.5 (18 ~ 43)	15.5 (11 ~ 28)	*p* < 0.001
Motor conduction test	Latency (ms)	4.72 ± 0.66	4.57 ± 0.74	*p* = 0.131
	Distal amplitude (mV)	9 ± 2.38	8.94 ± 2.06	*p* = 0.871
	Conduction velocity (m/s)	55.87 ± 5.64	54.67 ± 3.47	*p* = 0.414
Sensory conduction test	Latency (ms)	3.63 ± 0.68	3.27 ± 0.54	*p* = 0.043
	Amplitude (μV)	16.96 ± 9.7	21.04 ± 11.2	*p* = 0.02
	Conduction velocity (m/s)	36.93 ± 6.53	40.8 ± 6.62	*p* = 0.034
CMAP scan analysis	MScanFit MUNE	76.19 ± 26.49	91.31 ± 28.76	*p* < 0.001
	MScanFit Mean unit (μV)	118.52 ± 32.2	97.24 ± 25.39	*p* = 0.003
	StairFit MUNE	61.33 ± 18.08	58.72 ± 14.74	*p* = 0.4644
	StairFit Mean unit (μV)	137.19 ± 22.43	143.19 ± 20.94	*p* = 0.1964
	STEPIX	98.00 ± 31.05	95.50 ± 25.98	*p* = 0.6410
	AMPIX	90.67 ± 25.03	91.49 ± 24.17	*p* = 0.8099
	S0 (mA)	8.28 ± 3.3	6.94 ± 2.42	*p* = 0.203
	S100 (mA)	20.25 ± 7.2	16.44 ± 5.09	*p* = 0.095
	Stimulus range (mA)	11.75 (5.5 ~ 22)	9 (5.5 ~ 19)	*p* = 0.114

Mean ± standard deviation was used in the table. Median (data range) was reported for BCTQ and Stimulus range. CMAP, Compound Muscle Action Potential; MUNE, Motor Unit Number Estimation; CTS, Carpal Tunnel Syndrome; BCTQ, Boston Carpal Tunnel Questionnaire; STEPIX, step index; AMPIX, amplitude index.

## 4. Discussion

This study presents the application of shock wave therapy for treatment of CTS patients. The sudden release of energy from shock waves is capable of an instantaneous increase in pressure and high-speed wave conduction, which can also act on the deep muscle tissue. As a noninvasive treatment method, radial extracorporeal shock wave therapy has shown clinical efficacy in pain relief and functional improvement in treatment of bone and tendon diseases (Ioppolo et al., 2014). The usefulness of shock wave treatment on neurological disorders has also been reported (Manganotti and Amelio, 2005; Guo et al., 2022). Consistent to previous applications of shock waves in treating CTS (Wu et al., 2016; Xie et al., 2022), this study confirms that radial extracorporeal shock wave treatment has a definite therapeutic effect on CTS. The patients’ symptoms improved significantly after the treatment, as demonstrated by the favorable changes in BCTQ scale.

The mechanisms of extracorporeal shock wave therapy in CTS are not yet completely understood. As discussed in previous literature (Manganotti and Amelio, 2005; Ioppolo et al., 2014; Guo et al., 2022), the main mechanisms associated with the treatment effect most likely include metabolic and cavitation effects in the impact area as a result of the biological effects during high pressure to chemical signal transduction. The released growth factors and the facilitated inflammation resolution can promote microvascular neovascularization tissue regeneration and repair. Shock wave therapy was also reported to have an effect on peripheral nerve regeneration (Hausner and Nógrádi, 2013; Sağir et al., 2019). These may collectively promote the healing process of the injured area and improve symptoms.

Electrophysiological studies indicated that sensory nerve latency, wave amplitude and conduction velocity were improved, but no significant changes in motor conduction study parameters were observed after the treatment, suggesting that the improvement is mainly associated with sensory properties. One limitation of the nerve conduction study is that it may not be sensitive enough to detect early-stage or slowly progressing motor unit loss, due to muscle fiber reinnervation by still surviving motor units. Because of this, MUNE was performed in the current study which is more sensitive to axon loss. Previous MUNE studies reported decreased MUNE values in CTS patients including those who did not exhibit clinical signs or symptoms (Bayrak et al., 2007; Nashed et al., 2010; Sohn et al., 2011; Yilmaz et al., 2016). A primary feature of the current study was the application of the CMAP scan recording for assessing neuromuscular changes associated with the shock wave therapy, which has several advantages over the traditional MUNE methods applied to CTS patients. Compared with previous MUNE methods based on mean motor unit size estimation from a small sample of motor units (thus potentially leading to a biased mean size estimation), CMAP scan provides information about all motor units contributing to the maximum CMAP. Therefore, the MUNE derived from CMAP scan can avoid the inherent bias of extrapolating from a small sample of motor units. Additionally, both CMAP scan protocols and data processing can be fast and automatic, an important feature for clinical applications.

Three different methods were used to process CMAP scan data in this study, and unexpectedly achieved different results. MScanFit suggested an increased motor unit number after shock wave therapy treatment, while StairFit and STEPIX indicated that there was no significant difference in motor unit number before and after the treatment. A detailed analysis of different CMAP scan processing methods is beyond the scope of this brief research report. The reasons for the inconsistencies from different CMAP scan processing methods are certainly worthwhile targets for further investigation.

The main limitation of the present study is a lack of a control group receiving placebo therapy. Other limitations include relatively small number of subjects participating in the study, and lack of needle EMG examination. It would have been more informative to also record needle EMG to perform quantitative motor unit action potential analysis or single fiber EMG analysis. This could help provide more definite information about innervation/reinnervation changes associated with shock wave treatment for CTS patients.

## Data availability statement

The raw data supporting the conclusions of this article will be made available by the authors, without undue reservation.

## Ethics statement

The studies involving humans were approved by Institutional Review Board of Shanghai Jiao Tong University. The studies were conducted in accordance with the local legislation and institutional requirements. The participants provided their written informed consent to participate in this study.

## Author contributions

YZ, QX, and PZ: study design. YZ, HZ, and PX: data collection. YZ: MScanFit MUNE processing, writing—original draft preparation. MC: StairFit MUNE processing, STEPIX processing. YZ, HZ, PX, MC, QX, and PZ: data analysis and interpretation. PZ: writing—revision. HZ and QX: writing—review and editing. QX and PZ: study supervision. All authors have read and agreed to the published version of the manuscript.
